# Assessment of Cognitive Function in Patients After COVID-19 Infection

**DOI:** 10.1001/jamanetworkopen.2021.30645

**Published:** 2021-10-22

**Authors:** Jacqueline H. Becker, Jenny J. Lin, Molly Doernberg, Kimberly Stone, Allison Navis, Joanne R. Festa, Juan P. Wisnivesky

**Affiliations:** 1 Division of General Internal Medicine, Icahn School of Medicine at Mount Sinai, New York, New York; 2 Department of Neurology, Icahn School of Medicine at Mount Sinai, New York, New York; 3 The Catherine and Henry J. Gaisman Division of Pulmonary, Critical Care and Sleep Medicine, Icahn School of Medicine at Mount Sinai, New York, New York

## Abstract

This cross-sectional study examines rates of cognitive impairment among patients who survived COVID-19 and whether the care setting was associated with cognitive impairment rates.

## Introduction

People who have survived COVID-19 frequently complain of cognitive dysfunction, which has been described as brain fog. The prevalence of post–COVID-19 cognitive impairment and the association with disease severity are not well characterized. Previous studies on the topic have been limited by small sample sizes and suboptimal measurement of cognitive functioning.^1^ We investigated rates of cognitive impairment in survivors of COVID-19 who were treated in outpatient, emergency department (ED), or inpatient hospital settings.

## Methods

We analyzed data in this cross-sectional study from April 2020 through May 2021 from a cohort of patients with COVID-19 followed up through a Mount Sinai Health System registry. Study participants were 18 years or older, spoke English or Spanish, tested positive for SARS-CoV-2 or had serum antibody positivity, and had no history of dementia. Participant demographic characteristics (eg, age, race, and ethnicity) were collected via self-report. Cognitive functioning was assessed using well-validated neuropsychological measures: Number Span forward (attention) and backward (working memory), Trail Making Test Part A and Part B (processing speed and executive functioning, respectively), phonemic and category fluency (language), and the Hopkins Verbal Learning Test–Revised (memory encoding, recall, and recognition). The Mount Sinai Health System Institutional Review Board approved this study, and informed consent was obtained from study participants. The study followed the Strengthening the Reporting of Observational Studies in Epidemiology (STROBE) reporting guideline.

We calculated the frequency of impairment on each measure, defined as a *z* score of less than or equal to 1.5 SDs below measure-specific age-, educational level–, and sex-adjusted norms.^2,3^ Logistic regression assessed the association between cognitive impairment and COVID-19 care site (outpatient, ED, or hospital), adjusting for race and ethnicity, smoking, body mass index, comorbidities, and depression. The threshold for statistical significance was α = .05, and the tests were 2-tailed. Analyses were performed using SAS, version 9.4 (SAS Institute).

## Results

The mean (IQR) age of 740 participants was 49 (38-59) years, 63% (n = 464) were women, and the mean (SD) time from COVID-19 diagnosis was 7.6 (2.7) months (Table 1). Participants self-identified as Black (15%), Hispanic (20%), or White (54%) or selected multiracial or other race and ethnicity (11%; other race included Asian [4.5%, n = 33)] and those who selected “other” as race). The most prominent deficits were in processing speed (18%, n = 133), executive functioning (16%, n = 118), phonemic fluency (15%, n = 111) and category fluency (20%, n = 148), memory encoding (24%, n = 178), and memory recall (23%, n = 170; Table 2).

**Table 1.** Characteristics of Patients Who Had COVID-19

Characteristic	No. of Patients (%) (N = 740)
Time from diagnosis to baseline visit, mo, mean (SD)	7.6 (2.7)
Age, y, mean (SD)	49.0 (14.2)
Sex	
Female	464 (63)
Male	276 (37)
Race/ethnicity	
Black	112 (15)
Hispanic	149 (20)
White	397 (54)
Multiracial or other^a^	75 (11)
Educational level, y	
≤12	103 (14)
>12	636 (86)
Income	
<$25 000	109 (15)^b^
$25 000-$60 000	113 (15)
$60 000-$150 000	244 (33)
>$150 000	206 (28)
Former smoker	226 (31)
Comorbidities	
Hypertension	191 (26)
Diabetes	74 (10)
Asthma	179 (24)
Cancer	72 (10)
Body mass index^c^	
Normal weight	273 (37)^b^
Overweight	212 (29)
Obese	249 (34)
Site of COVID-19 care	
Outpatient	379 (51)
Emergency department	165 (22)
Hospital	196 (27)

^a^ Other included Asian (33 [4.5%]), and the remainder included those who reported other as race.

^b^ The sum of the subcategories is less than 100 due to missing data.

^c^ Calculated as weight in kilograms divided by height in meters squared. Normal weight is a body mass index of 18.5 to 24.9, overweight is 25.0 to 30.0, and obese is greater than 30.0.

**Table 2.** Prevalence of Cognitive Impairment After COVID-19 Infection

Cognitive domain	Impaired (*z* score ≤1.5), No. (%)				Adjusted odds ratio (95% CI)^a^	
	Total (N = 740)	Outpatient (n = 379)	ED (n = 165)	Hospitalized (n = 196)	ED vs outpatient	Hospital vs outpatient
Attention	74 (10)	19 (5)	10 (6)	29 (15)	0.8 (0.3-2.0)	2.8 (1.3-5.9)
Working memory	74 (10)	30 (8)	17 (10)	29 (15)	1.0 (0.5-2.2)	1.7 (0.8-3.3)
Processing speed	133 (18)	57 (15)	21 (13)	55 (28)	0.7 (0.4-1.3)	1.4 (0.8-2.5)
Executive functioning	118 (16)	45 (12)	23 (14)	53 (27)	1.0 (0.5-1.8)	1.8 (1.0-3.4)
Phonemic fluency	111 (15)	42 (11)	25 (15)	39 (20)	0.9 (0.5-1.8)	1.5 (0.8-2.8)
Category fluency	148 (20)	49 (13)	35 (21)	69 (35)	1.8 (1.1-3.1)	3.0 (1.7-5.2)
Memory encoding	178 (24)	61 (16)	43 (26)	73 (37)	1.7 (1.0-3.0)	2.3 (1.3-4.1)
Memory recall	170 (23)	45 (12)	38 (23)	76 (39)	1.5 (0.9-2.6)	2.2 (1.3-3.8)
Memory recognition	74 (10)	34 (9)	20 (12)	25 (13)	1.5 (0.8-3.0)	1.1 (0.5-2.4)

Abbreviation: ED, emergency department.

^a^ Adjusted for race and ethnicity, smoking history, body mass index (calculated as weight in kilograms divided by height in meters squared), comorbidities, and depressive symptoms.

In adjusted analyses, hospitalized patients were more likely to have impairments in attention (odds ratio [OR]: 2.8; 95% CI: 1.3-5.9), executive functioning (OR: 1.8; 95% CI: 1.0-3.4), category fluency (OR: 3.0; 95% CI: 1.7-5.2), memory encoding (OR: 2.3; 95% CI: 1.3-4.1), and memory recall (OR: 2.2; 95% CI: 1.3-3.8) than those in the outpatient group. Patients treated in the ED were more likely to have impaired category fluency (OR: 1.8; 95% CI: 1.1-3.1) and memory encoding (OR: 1.7; 95% CI: 1.0-3.0) than those treated in the outpatient setting. No significant differences in impairments in other domains were observed between groups.

## Discussion

In this study, we found a relatively high frequency of cognitive impairment several months after patients contracted COVID-19. Impairments in executive functioning, processing speed, category fluency, memory encoding, and recall were predominant among hospitalized patients. The relative sparing of memory recognition in the context of impaired encoding and recall suggests an executive pattern. This pattern is consistent with early reports describing a dysexecutive syndrome after COVID-19^4^ and has considerable implications for occupational, psychological, and functional outcomes. It is well known that certain populations (eg, older adults) may be particularly susceptible to cognitive impairment after critical illness^5^; however, in the relatively young cohort in the present study, a substantial proportion exhibited cognitive dysfunction several months after recovering from COVID-19. The findings of this study are generally consistent with those of research on other viruses (eg, influenza).^6^

Limitations of this study include a potential sampling bias, as some participants may have presented to Mount Sinai Health System because of health concerns. Future studies should investigate long-term post–COVID-19 cognitive trajectories and the association with neuroimaging findings to assess potential mechanisms.

## Conclusions

The association of COVID-19 with executive functioning raises key questions regarding patients’ long-term treatment. Future studies are needed to identify the risk factors and mechanisms underlying cognitive dysfunction as well as options for rehabilitation.
